# Folate receptor alpha in platinum-resistant ovarian cancer: prevalence in a multicenter Polish cohort and review of the evidence

**DOI:** 10.1007/s12094-025-04179-3

**Published:** 2025-12-22

**Authors:** Jędrzej Borowczak, Justyna Durślewicz, Monika Durzyńska, Jakub Cheliński, Maria Filipek, Marek Zdrenka, Łukasz Szylberg

**Affiliations:** 1 Faculty of Medicine, Bydgoszcz University of Science and Technology, Aleje Prof. S. Kaliskiego 7, 85-796 Bydgoszcz, Poland; 2 Clinical Department of Oncology, Oncology Centre - Prof. Franciszek Łukaszczyk Memorial Hospital, 85-796 Bydgoszcz, Poland; 3 Department of Tumor Pathology and Pathomorphology, Oncology Centre - Prof. Franciszek Łukaszczyk Memorial Hospital, 85-796 Bydgoszcz, Poland; 4 Department of Pathology, Maria Sklodowska-Curie National Research Institute of Oncology, 02-781 Warsaw, Poland; 5 Department of Obstetrics, Gynaecology and Oncology, Ludwik Rydygier Collegium Medicum in Bydgoszcz, Nicolaus Copernicus University in Toruń, Bydgoszcz, Poland

**Keywords:** FOLR1, FRα, Epidemiology, Platin-resistant, Ovarian cancer, Mirvetuximab

## Abstract

**Background:**

Folate receptor alpha (FRα, FOLR1) is the required biomarker for mirvetuximab soravtansine, a treatment option for patients with platinum-resistant ovarian cancer. Since its approval, the drug has been introduced into national drug programs, but prevalence data from the Polish population are lacking. This study provides the first multicenter assessment of FOLR1 expression in Polish ovarian cancer in the context of treatment eligibility.

**Methods:**

We retrospectively analyzed two institutional cohorts comprising 229 epithelial ovarian cancer cases from the Oncology Center in Bydgoszcz and the National Institute of Oncology in Warsaw. Immunohistochemistry was performed on formalin-fixed, paraffin-embedded tissue using the VENTANA FOLR1 (FOLR1-2.1) RxDx Assay on the BenchMark ULTRA platform. Membranous staining of moderate or strong intensity in ≥ 75% of viable tumor cells was considered positive, in accordance with criteria for FOLR1-targeted therapy.

**Results:**

Positive FOLR1 expression was observed in 116 of 229 (50.7%, 95% CI 44.2–57.2) analyzed tumors, with 40% showing high expression. In most of these cases, staining intensity was moderate to strong, meeting the established threshold for therapeutic eligibility. In ~ 18% of tumors, FOLR1 expression fell into the borderline range (65–85%).

**Conclusion:**

Approximately half of the ovarian cancer patients in this multicenter Polish cohort demonstrated FOLR1 positivity. Although this prevalence is lower than that reported in high-grade serous carcinoma cohorts, it likely reflects histological heterogeneity and methodological differences between studies. These findings provide the first population-based evidence from Poland and support the implementation of FOLR1 testing to guide access to mirvetuximab soravtansine. Further research integrating clinical and molecular data is warranted to validate these results and assess implications for cost-effectiveness and patient outcomes.

**Supplementary Information:**

The online version contains supplementary material available at 10.1007/s12094-025-04179-3.

## Introduction

The folate receptor alpha (FOLR1, FRα) is a glycoprotein anchored to the cell membrane via a glycosylphosphatidylinositol (GPI) moiety. It mediates high-affinity folate uptake and, among the three main isoforms (FRα, FRβ, FRγ) is the predominant receptor in epithelial tissues [1–3]. In normal conditions, FOLR1 expression is restricted mainly to luminal epithelia of the female reproductive tract, kidney, lung, and salivary glands, which limits direct exposure to circulating folates [3].

Folate compounds are essential cofactors in purine and pyrimidine biosynthesis, DNA methylation, and one-carbon metabolism [2, 3]. Their role in supporting proliferation provides biological rationale for the marked overexpression of FOLR1 in malignant cells, particularly in non-mucinous ovarian tumors [3, 4]. Elevated FOLR1 levels in tumor tissue and serum have been consistently reported in patients with ovarian cancer compared to healthy controls, supporting its role both as a biomarker and a mediator of tumor biology [4, 5].

The restricted distribution of FOLR1 in healthy tissues and its upregulation in ovarian carcinoma have established it as a promising diagnostic and therapeutic target. Over the past decade, several therapeutic approaches have been investigated, including folate-conjugated cytotoxics, monoclonal antibodies, and antibody–drug conjugates (ADCs). Among these, mirvetuximab soravtansine has demonstrated the strongest evidence to date. In the phase III MIRASOL trial, mirvetuximab improved both progression-free and overall survival in women with platinum-resistant ovarian cancer whose tumors expressed high levels of FOLR1 [6]. On this basis, the drug received regulatory approval in the United States and Europe, accompanied by a companion diagnostic assay (VENTANA FOLR1 RxDx Assay) to standardize patient selection. Currently, the efficacy of mirvetuximab with bevacizumab compared to bevacizumab alone is being tested in the GLORIOSA phase III clinical trials as maintenance for patients with FRα-positive recurrent platinum-sensitive epithelial ovarian cancer [7].

Nevertheless, population-specific data on FOLR1 prevalence remains scarce and heterogeneous. Most reports come from large international consortia or single-institution studies in North America and Western Europe [8, 9]. Reported prevalence varies substantially by histological type, assay method, and scoring criteria, complicating cross-study comparison. For Central and Eastern Europe, including Poland, robust epidemiological data are lacking. This knowledge gap is particularly concerning given the anticipated inclusion of mirvetuximab in national drug programs, where the proportion of eligible patients directly influences cost-effectiveness analyses and resource allocation.

Here, we present the first systematic evaluation of FOLR1 expression in a large, treatment-refractory cancer cohort from tertiary, comprehensive cancer center in Poland. Our findings provide population-specific prevalence estimates and may inform clinical implementation and health policy decisions regarding access to FOLR1-targeted therapy.

## Materials and methods

### Study cohort

The analysis included the largest Polish cohort published to date, comprising 205 platinum-resistant epithelial ovarian cancer cases (both institutional and consultative material) submitted for FOLR1 testing and evaluated at the Oncology Center in Bydgoszcz. In addition, we also included an independent validation cohort of 24 epithelial ovarian cancer cases tested at the National Institute of Oncology in Warsaw.

### Immunohistochemistry

FOLR1 expression was assessed by immunohistochemistry on formalin-fixed, paraffin-embedded tissue sections using the VENTANA FOLR1 (FOLR1-2.1) RxDx Assay (Ventana/Roche, Tucson, AZ, USA) [10]. This clinically validated test is designed to identify patients with ovarian, fallopian tube, or primary peritoneal carcinoma eligible for FOLR1-targeted therapy (ELAHERE). Immunostaining was performed on the BenchMark ULTRA automated platform with the OptiView DAB IHC Detection Kit, using the FOLR1-2.1 antibody clone provided in the assay kit.

### Staining interpretation

In epithelial ovarian tumors stained with anti-FOLR1 antibody (clone 2.1), both membranous and cytoplasmic localization of the immunohistochemical signal may be observed. For the purpose of FOLR1 status assessment, only membranous staining in tumor cells is considered. It was defined as membrane-localized staining, including both apical patterns (on the luminal surface of tumor cells) and circumferential patterns (encircling the entire cell membrane). Cytoplasmic signal—typically resulting from endosomal antigen storage or nonspecific background staining—is a known phenomenon with the FOLR1-2.1 antibody but is not taken into account in result interpretation. Incomplete patterns, such as dot-like or partial staining, are also acceptable, provided the signal is clearly localized along the membrane contour of tumor cells.

### Scoring system

**Fig. 1:**
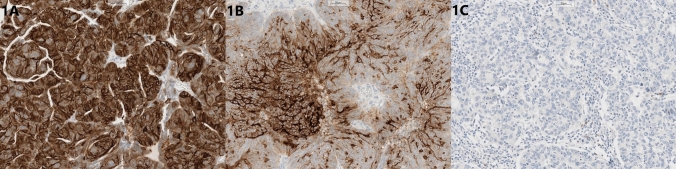
Representative FOLR1 immunohistochemical staining showing **A** high, **B** moderate, and **C** negative expression

The immunohistochemical evaluation of FOLR1 expression was semi-quantitative, incorporating both the percentage of tumor cells with positive membranous staining and the staining intensity. A four-tier scoring system is applied: (0)—no staining; (1+)—weak, barely perceptible membranous staining; (2+)—moderate intensity, comparable to a reference tissue (e.g., fallopian tube epithelium); (3+)—strong, intense membranous signal, clearly exceeding the background and moderate reference (Fig. 1). A case was considered positive (FOLR1+) when ≥ 75% of tumor cells demonstrate membranous staining of moderate or strong intensity (2+ and/or 3+). Cases with < 75% positive tumor cells were classified as FOLR1-negative (low or no expression). This threshold distinguishing patients likely to benefit from targeted anti-FOLR1 therapy [6]. For descriptive analyses, FOLR1 expression was stratified into six categories: 0% (no expression), 1–25% (low expression), 26–50% (moderate expression), 51–64% (marked expression, not yet diffuse), 65–85% (borderline expression), and > 85% (high/diffuse expression).

### Quality control and diagnostic validity

To be considered diagnostically valid, a specimen was required to contain at least ~ 100 viable tumor cells. Samples with low cellularity were deemed unreliable and were not reported. The presence of an internal positive control (e.g., normal fallopian tube epithelium) was recommended to confirm technical adequacy of the staining and to calibrate intensity interpretation, as this tissue typically demonstrates moderate apical membranous staining. Cases in which technical artifacts obscured staining or the number of viable tumor cells was insufficient (< 100), the result were classified as non-diagnostic. The manufacturer further recommends that borderline cases—those with positive cell percentages close to the 75% cutoff (e.g., 65–85%)—should be reviewed by a second, and if needed, a third pathologist to ensure accuracy [10].

### Statistical analysis

All statistical analyses were performed using Statistica version 13.3 (Statsoft) and Microsoft Excel 2019. Proportions were presented together with 95% confidence intervals (Wilson method). Differences in FOLR1 positivity between the Bydgoszcz and Warsaw cohorts were assessed using Fisher’s exact test. A two-sided *p* < 0.05 was considered statistically significant .

## Results

**Fig. 2:**
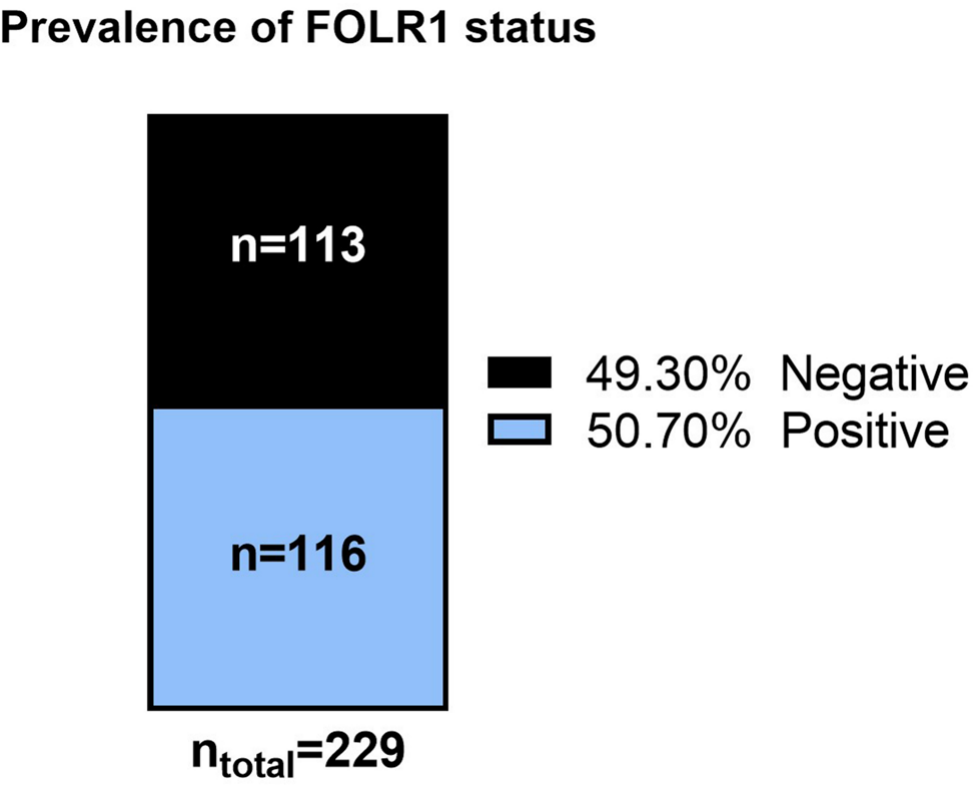
Prevalence of positive FOLR1 expression in the Polish cohort

**Fig. 3:**
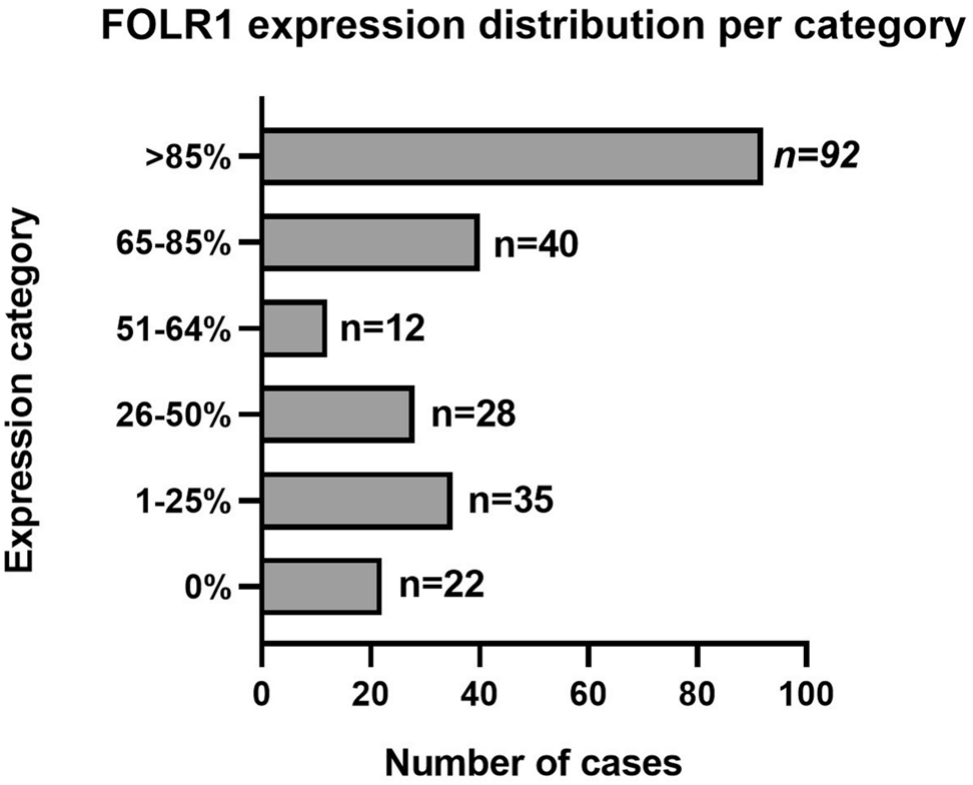
FOLR1 expression distribution per category

The multicenter Polish cohort comprised 229 epithelial ovarian cancer cases, of which 116 (50.7%, 95% CI 44.2–57.2) showed positive FOLR1 expression (Fig. 2). This dataset constitutes the largest Polish dataset on FOLR1 expression to date. Among the 229 evaluable cases, 9.6% showed no FOLR1 expression, 15.3% low expression (1–25%), 12.2% moderate (26–50%), 5.2% marked (51–64%), 17.5% borderline (65–85%), and 40.2% high expression (> 85%) (Fig. 3, Supplementary Table 1). The majority of positive cases across both institutions showed intermediate to strong membranous staining (2+/3+), fulfilling the diagnostic threshold for therapeutic eligibility. There was no significant difference in FOLR1 positivity between the Bydgoszcz and Warsaw cohorts (*p* = 0.63).

## Discussion

In our analysis, 116 of 229 (50.7%) ovarian cancer cases demonstrated positive FOLR1 expression. Most of the evaluated cases showed intermediate or strong FOLR1 expression, meeting the inclusion criteria for mirvetuximab soravtansine. Since the EMA approval of mirvetuximab soravtansine in 2024, no studies have evaluated the prevalence of FOLR1 positivity in the Polish population. Our findings provide the first insight into the potential size of the eligible patient group. However, they warrant further contextualization and should be interpreted in relation to data from other international cohorts, contrasted with results from other populations, and critically examined for potential methodological or biological factors that may have influenced the observed prevalence.

FOLR1 expression varies substantially between studies and histological subtypes of epithelial ovarian cancer, ranging from ~ 20 to 25% in endometrioid to over 40% in clear cell carcinomas, while being largely absent in mucinous tumors. High-grade serous carcinoma (HGSC) remains the most relevant in clinical practice, with FOLR1+ observed in 40–45% of cases. However, these estimates are based on heterogeneous international cohorts, and reliable subtype-specific prevalence data for the Polish population are lacking (Table 1). In the OTTA consortium study, 76.2% of high-grade serous carcinomas (HGSC) were FOLR1-positive, whereas expression was much lower in mucinous carcinomas (11%) and inconsistently reported in clear cell carcinoma. In this study, cases were positive for FOLR1 if strong FOLR1 staining was detected in ≥ 1% of tumor cells [8]. Kalli et al. reported FOLR1+ in 72% of primary ovarian cancers and 81.5% of recurrences, with positive expression persisting at metastatic sites; however, they defined FOLR1 positivity as any detectable FRα staining in ≥ 1 tumor cell in any sample from an individual [9]. Rushton et al. analyzed over 5000 HGSC cases and found that HGSC had higher FOLR1+ prevalence than low grade serous carcinomas (LGSC; 43.5% vs 24/6%, respectively). Tumors with ≥ 75% of viable tumor cells with moderate or strong staining (2+ or 3+) were considered FOLR-1 positive. Importantly, in both HGSC and LGSC, FOLR1 positivity was associated with improved median survival (~ 98 vs. ~ 87 months, respectively) [11]. Liang et al. also reported FOLR1+ in 76.25% of all ovarian cancers, with higher expression in more advanced FIGO stages (III–IV vs. I–II; *p* = 0.038) [12]. The discrepancies may partly reflect methodological heterogeneity. While the RxDx companion diagnostic defines FOLR1+ as ≥ 75% of tumor cells with ≥ 2+ membranous staining, earlier studies used different antibodies and scoring systems (e.g. H-score, lower cut-offs, or presence of any FOLR1+ cells) [8, 9, 12]. In our multicenter dataset the prevalence of FOLR1 positivity was reached 50.7%, supporting the robustness of the findings in the Polish population. However, discrepancies between international studies and methodological heterogeneity still complicate cost-effectiveness analyses of mirvetuximab implementation, as the true proportion of eligible patients remains to be defined in individual populations.

**Table 1:** Prevalence of FOLR1+ ovarian cancers across studies

EOC subtype	Study, year	Cases, *n*	FOLR1+ (%)	Definition of FOLR1 positivity	Description
HGSC	Kalli et al., 2008 [9]	186 + 27 recurrences	72% primary, 81.5% recurrence	Any detectable FRα staining in ≥ 1 tumor cell in any sample from an individual	Similar FOLR1+ rates in primary and metastatic sites; FOLR1+ persisted in recurrences
	Köbel et al. (OTTA), 2014 [8]	1507	76.2%	Strong FOLR1 staining in ≥ 1% of tumor cells	Higher 2-year OS in FOLR1+ tumors
	Rushton et al., 2024 [11]	5086	43.5%	≥ 75% of viable tumor cells with moderate or strong staining (2+ or 3+)	FOLR1+ was associated with longer median OS
LGSC	Rushton et al., 2024 [11]	281	24.6%	≥ 75% of viable tumor cells with moderate or strong staining (2+ or 3+)	FOLR1+ was associated with longer median OS
	Köbel et al. (OTTA), 2014 [8]	91	49.5%	Strong FOLR1 staining in ≥ 1% of tumor cells	No correlation between FOLR1 status and patients survival
EC	Köbel et al. (OTTA), 2014 [8]	564	29.4%	Strong FOLR1 staining in ≥ 1% of tumor cells	No correlation between FOLR1 status and patients survival
CCC	Köbel et al. (OTTA), 2014 [8]	259	31.6%	Strong FOLR1 staining in ≥ 1% of tumor cells	FOLR1+ in CCC was associated with shorter PFS irrespective of follow-up time, especially in FIGO stage I/II tumors
Mucinous	Köbel et al. (OTTA), 2014 [8]	193	11.4%	Strong FOLR1 staining in ≥ 1% of tumor cells	Lower FOLR1+ prevalence compared to other subtypes

*EOC* epithelial ovarian cancer, *HGSC* high-grade serous carcinoma, *LGSC* low-grade serous carcinoma, *EC* endometrioid carcinoma, *CCC* clear cell carcinoma, *OS* overall survival, *PFS* progression-free survival, *FIGO* International Federation of Gynecology and Obstetric

It is important to note that ~ 18% of tumors showed FOLR1 expression in the borderline range (65–85%). According to the companion diagnostic manual, such cases required review by a second, and if necessary by a third pathologist to confirm FOLR1 status [10]. This reflects the intratumoral heterogeneity and uneven distribution of FOLR1, and serves as a validation mechanism intended to prevent misclassification of borderline cases [13, 14]. In larger retrospective series, primary ovarian tumors were more likely to be FOLR1 positive than metastases, while in patients with multiple evaluated specimens results were discordant in 37.5% of cases [13]. Martin et al. also reported that the concordance between archival and biopsy samples reached only ~ 70%, although the expression levels between paired pre- and post-treatment biopsies were stable [14]. These findings highlight both spatial and temporal variability in FOLR1 assessment, supporting careful sampling strategies in addition to pathologist adjudication of borderline cases.

The issue of re-sampling in tumors with heterogeneous or borderline FOLR1 expression (65–85%) remains unresolved [10]. Evidence on intratumoral heterogeneity is limited, and to date, no guidelines provide recommendations on whether additional blocks from the same tumor should be routinely tested [14, 15]. Clinicians often raise the question whether such strategies could increase the proportion of patients meeting eligibility criteria, but there are no data showing that discordant cases benefit from mirvetuximab to the same extent as those with uniformly high expression [6]. At present, pathologist adjudication in borderline cases remains the only standardized safeguard against misclassification.

A key observation from our analysis is the wide variation in FOLR1 expression across epithelial ovarian cancer histotypes, reflecting the high biological heterogeneity of this disease. In published datasets, high-grade serous carcinoma shows the highest FOLR1 expression at 43.5% using standardized criteria [11]. In comparison, our cohort demonstrated a 50.7% FOLR1-positive rate, which is consistent with the expected HGSC-dominant case mix. By contrast, LGSC shows substantially lower FOLR1 expression (24.6% per Rushton, 49.5% with less stringent criteria), while other histotypes demonstrate significantly lower or absent FOLR1 expression [8, 9, 11]. Equally important, the near-equal proportion of FOLR1-negative tumors (49.3%) highlights that platinum resistance arises through multiple parallel mechanisms, and that FOLR1 expression captures only one of several clinically relevant pathways.

Beyond histotype variation, cohort characteristics and population-specific factors may also account for the observed discrepancies [10]. Tumor biology, including biological markers expression varies according to race, ethnicity, age, or sex, which may be mediated by genetic/epigenetic differences and socio-economic disparities and affect the outcomes of ovarian cancer patients [16]. For instance, Smith et al. showed that Black and American Indian women were less likely to have elevated CA-125 level at diagnosis compared to White women, suggesting a risk of underdiagnosis and delayed treatment in these groups [17]. Since we currently lack data regarding the disparities in FOLR1 expression between various populations, further research is needed.

While disparities in marker expression highlight the importance of demographic context, our analysis focused on relatively homogenous Polish cohort of platinum-resistant, advanced cases. All institutional and consultative ovarian cancer specimens were newly evaluated for FOLR1 expression. However, due to the nature of the data, part of the information on the timing of tissue sampling (e.g. whether derived from primary or post-treatment specimens) is lacking. While some authors found that FOLR1 expression persists after chemotherapy, others reported that FOLR1 levels are actually lower in platinum-resistant tumors compared to sensitive ones [18–20]. This uncertainty regarding temporal changes in FOLR1 expression highlights the need for dynamic evaluation of FOLR1 levels before and after treatment in the Polish population.

Several biological mechanisms have been proposed to explain a potential interplay between FOLR1 expression and platinum resistance. High FOLR1 levels may increase folate uptake and one-carbon metabolism, mitigating platinum-induced DNA damage by supporting nucleotide synthesis and DNA repair pathways [3]. Dysregulation of folate-dependent enzymes such as methionine synthase reductase (MTRR) has also been associated with cisplatin resistance through disturbing apoptotic and autophagic pathways; in preclinical models, MTRR silencing partially reversed this phenotype [21]. In addition, FOLR1 may promote pro-survival ERK1/2 and JAK-STAT3 signaling, independently contributing to reduced sensitivity to chemotherapy [22, 23].

Conversely, platinum-resistant tumors downregulate FOLR1 as part of dedifferentiation or metabolic reprogramming. Platinum-resistant tumors are also enriched for cancer stem-cell populations with hedgehog pathway activation and altered metabolic demands, which may favour either FOLR1-positive or FOLR1-negative phenotypes depending on the predominant resistance mechanism [24]. However, current evidence is inconsistent. While Huang et al. demonstrated significantly lower FOLR1 levels in platinum-resistant ovarian cancer cells than platinum-sensitive ones, Rubinsak et al. showed that FOLR1 is expressed in 100% of platinum-resistant ovarian cancer samples, suggesting selection of chemotherapy-resistant clones [18, 20]. Overall, current evidence indicates that FOLR1 may contribute to, but not solely define the biology of platinum resistance.

Despite these uncertainties, our results help fill a significant gap in epidemiological data on FOLR1 expression in the Polish population—approximately half of patients with platinum-resistant epithelial ovarian cancer met the criteria for FOLR1+ status and may therefore benefit from mirvetuximab therapy. Such findings facilitate resource allocation ahead of the anticipated introduction of the national drug program in October 2025. By providing real-world epidemiological data, our results may help inform cost-effectiveness modelling in Poland; however, further research is required to validate these findings and to address unresolved clinical scenarios, such as outcomes after prior PARP inhibitor exposure, BRCA and HRD status, and the impact of multiple prior chemotherapy lines.

Several technical limitations also need to be addressed. First, the retrospective nature of our study introduces potential selection bias and limits the strength of the conclusions. Second, detailed clinical data, including BRCA mutation status, prior exposure to PARP inhibitors, and histopathological subtype stratification, were not available, which prevented more detailed correlative analyses. It is important to note that all FOLR1 tests included in this study were requested as part of the early-access pathway for mirvetuximab soravtansine, which at the time represented the only therapeutic option for patients with platinum-resistant epithelial ovarian cancer in Poland. In clinical practice, such requests are made almost exclusively for high-grade serous carcinomas. Although formal histotype annotation was not uniformly available in our retrospective dataset, the clinical context of FOLR1 testing strongly suggests that the cohort reflects a high-grade serous–dominant, platinum-resistant population. Third, only tumor samples were evaluated—there were no correlations to treatment outcomes. Finally, our findings require validation in independent and larger cohorts to confirm their generalizability in the Polish population. Nevertheless, as the largest dataset on FOLR1 expression in Polish ovarian cancer patients to date, this analysis provides a valuable reference point for future studies.

## Conclusions

This multicenter study provides the first epidemiological data on FOLR1 expression in the Polish population of patients with platinum-resistant epithelial ovarian cancer. Approximately half of the tumors met the criteria for FOLR1 positivity, suggesting that a large part of patients may benefit from mirvetuximab soravtansine therapy. These findings may help inform future cost-effectiveness analyses and resource allocation in the context of the planned national drug program and support policy makers in planning reimbursement strategies, but further validation integrating clinical and molecular data is needed. In addition, the observed borderline and heterogeneous expression patterns underscore the need for standardized diagnostic approaches and prospective studies linking FOLR1 status with treatment outcomes.

## Supplementary Information

Below is the link to the electronic supplementary material.

Supplementary file1 (DOCX 1641 KB)

## Data Availability

The data presented in this study are available on request from the Corresponding Author.
