# Supplementary material for: Folate receptor alpha in platinum-resistant ovarian cancer: prevalence in a multicenter Polish cohort and review of the evidence
Source: Clin Transl Oncol. 2025 Dec 22;28(6):2143–9. doi: 10.1007/s12094-025-04179-3 (PMC13186900; doi:10.1007/s12094-025-04179-3)
Supplement: Supplementary file 1 — Supplementary file1 (DOCX 1641 KB) [file 12094_2025_4179_MOESM1_ESM.docx]

**Supplementary Table 1.** Distribution of FOLR1 expression categories in the study cohort

| Expression range | Category | No. of cases | % of total (n=229) |
| --- | --- | --- | --- |
| 0% | No expression | 22 | 9.6% |
| 1-25% | Low expression | 35 | 15.3% |
| 26-50% | Moderate expression | 28 | 12.2% |
| 51-64% | Marked expression | 12 | 5.2% |
| 65-85% | Borderline expression | 40 | 17.5% |
| >85% | High expression | 92 | 40.2% |
